# Mesenchymal stem cell contact promotes *CCN1* splicing and transcription in myeloma cells

**DOI:** 10.1186/1478-811X-12-36

**Published:** 2014-06-25

**Authors:** Julia Dotterweich, Regina Ebert, Sabrina Kraus, Robert J Tower, Franz Jakob, Norbert Schütze

**Affiliations:** 1 Orthopedic Center for Musculoskeletal Research, Orthopedic Department, University of Würzburg, Brettreichstrasse 11, 97074 Würzburg, Germany; 2 Department of Internal Medicine II, University Hospital of Würzburg, Josef-Schneider Str. 2, 97080 Würzburg, Germany; 3 Section Biomedical Imaging, Department of Diagnostic Radiology, University Hospital Schleswig-Holstein, Campus Kiel, Germany

**Keywords:** CCN1, Multiple myeloma, Mesenchymal stem cells, Splicing

## Abstract

CCN family member 1 (CCN1), also known as cysteine-rich angiogenic inducer 61 (CYR61), belongs to the extracellular matrix-associated CCN protein family. The diverse functions of these proteins include regulation of cell migration, adhesion, proliferation, differentiation and survival/apoptosis, induction of angiogenesis and cellular senescence. Their functions are partly overlapping, largely non-redundant, cell-type specific, and depend on the local microenvironment. To elucidate the role of CCN1 in the crosstalk between stromal cells and myeloma cells, we performed co-culture experiments with primary mesenchymal stem cells (MSC) and the interleukin-6 (IL-6)-dependent myeloma cell line INA-6. Here we show that INA-6 cells display increased transcription and induction of splicing of intron-retaining *CCN1* pre-mRNA when cultured in contact with MSC. Protein analyses confirmed that INA-6 cells co-cultured with MSC show increased levels of CCN1 protein consistent with the existence of a pre-mature stop codon in intron 1 that abolishes translation of unspliced mRNA. Addition of recombinant CCN1-Fc protein to INA-6 cells was also found to induce splicing of *CCN1* pre-mRNA in a concentration-dependent manner. Only full length CCN1-Fc was able to induce mRNA splicing of all introns, whereas truncated recombinant isoforms lacking domain 4 failed to induce intron splicing. Blocking RGD-dependent integrins on INA-6 cells resulted in an inhibition of these splicing events. These findings expand knowledge on splicing of the proangiogenic, matricellular factor CCN1 in the tumor microenvironment. We propose that contact with MSC-derived CCN1 leads to splicing and enhanced transcription of *CCN1* which further contributes to the translation of angiogenic factor CCN1 in myeloma cells, supporting tumor viability and myeloma bone disease.

## Background

CCN family member 1 (CCN1) belongs to the modular extracellular matrix (ECM)-associated CCN protein family named according to three prototypic members (cysteine-rich angiogenic inducer 61 CYR61/CCN1, connective tissue growth factor CTGF/CCN2, and nephroblastoma overexpressed NOV/CCN3). Proteins share 30-50% primary sequence homology and are organized into four discrete domains with sequence relationships to insulin-like growth factor binding proteins (IGFBPs), the von Willebrand factor type C (vWC) repeat domain, the thrombospondin type I repeat (TSR) domain, and a carboxy-terminal (CT) domain containing a cysteine-knot motif [1]. These immediate early gene-encoded proteins are regulated by growth factors and hormones (e.g. transforming growth factor-beta (TGFβ), tumor-necrosis factor alpha (TNFα)), 1,25-dihydroxyvitamin D3, and mechanical forces [2-4]. Their functions are partly overlapping, yet appear to be mostly non-redundant, and are expressed in a cell-type specific manner dependent on the local microenvironment. CCN1 shows complex functionality due to specific interactions with a variety of binding partners including integrins and proteoglycans [5]. *CCN1* knockout is embryonically lethal in many pups due to alteration of chorioallantoic fusion, whereas most perish due to hemorrhage between E11.5 and E14.5 with only a very few being born alive, but dying within 24 h [6]. The multiple functions of CCN1 include regulation of cell migration, adhesion, proliferation, differentiation and survival/apoptosis, cellular senescence and ECM protein synthesis [4,7-10]. Through these diversity of functions, CCN1 modulates important biological processes including developmental processes, angiogenesis and tissue regeneration, and plays a role in pathological conditions such as wound healing, vascular diseases, inflammation, fibrosis and tumor development [10,11]. *CCN1* has been shown to act both as an oncogene, e.g. in mammary cancer, and as a tumor suppressor [10,12,13]. The importance of CCN1 in tumorigenesis originates from its diverse molecular functions which influence tumor development and metastasis by modulating angiogenesis, epithelial mesenchymal transition (EMT), and anoikis resistance [14]. Expression of CCN1 in tumors is characterized by deregulated protein levels, either of full length or truncated isoforms, whose diversity is expanded by post-translational processing, as well as by alternative splicing [15,16]. In the case of breast cancer tissue, alternative splicing of intron 3 has been linked to tumor progression and was regulated in tumor cells by exposure to hypoxic and acidic microenvironments [16-18]. In order to explain the apparent mismatch between the number of genes (25,000) versus the number of proteins that exist in humans (90,000), the last decade has seen extensive research about mechanisms that underlie the complexity of the proteome such as posttranslational modification mechanisms and alternative splicing. Splicing regulatory factors are currently under intensive research as “oncogenic alternative splicing switches” which may serve as promising new treatment targets in oncology [16]. In this context alternative splicing as a means of producing a biological diversity of CCN proteins has been discussed [15].

Multiple Myeloma (MM) is a B-cell malignancy characterized by clonal proliferation of a terminally-differentiated plasma cell, and is associated with immunoglobulin light chain or heavy chain production. MM is the second most common hematological malignancy and is accompanied by a high mortality and morbidity, despite recent progress in treatment modalities [19,20]. A major devastating complication of this disease is myeloma bone disease, which is associated with increased mortality and is driven by interaction between myeloma cells and cells of the bone marrow microenvironment, leading to imbalanced bone remodeling [21]. Bone metabolism is orchestrated by a complex interaction between bone-forming mesenchymal stem cells (MSC) and their osteogenic offspring (osteoblasts/osteocytes), and monocyte-derived osteoclasts. Bone formation is driven by at least three major osteogenic pathways, namely parathyroid hormone 1 receptor (PTH1R), bone morphogenetic proteins (BMP) and their receptors, and the wnt/frz/LRP5/6 pathway, along with sclerostin (SOST) and dickkopf-1 (DKK-1) which act as potent inhibitors of the wnt pathway [22]. Osteoclast development is dependent on colony-stimulating factors and receptor activator of NFĸB ligand (RANKL) produced by osteoblasts [23]. Myeloma cells also produce RANKL or RANKL-inducing molecules thereby initiating the classical vicious cycle of tumor-initiated osteolysis [24]. Myeloma bone disease dissociates bone resorption from bone formation and disintegrates the cooperation between osteogenic differentiation and angiogenesis. This supports myeloma progression and results in the inhibition of bone regeneration [25]. Bone regeneration is inhibited by myeloma-associated secreted inhibitors like DKK-1, secreted frizzled related proteins 2 and 3 (SFRP2/3), SOST and activin which interfere with osteogenic differentiation pathways [26,27]. Conversely, osteoblasts, for largely unknown reasons, can inhibit myeloma cell growth, as has been shown for MM treatment with the proteasome inhibitor bortezomib, which supports osteoblast function via enhanced vitamin D receptor signaling [28,29]. Antibody and antagonist-based neutralization strategies of osteogenic inhibitors (DKK-1, activin, SOST) are currently under development for the management of myeloma bone disease [30-33].

Myeloma cells are dependent on and influenced by stromal support. MSC have been described both as efficient supporters for myeloma cell growth and survival, and as potent disease modifiers that can inhibit MM cell growth [34]. MSC from MM patients exhibited intrinsic defects in osteogenic differentiation, which may permissively support MM expansion [35]. Moreover, overproduction of e.g. GDF15 by MSC, as seen in MM patients, is a powerful protective mechanism for MM cells and is linked to patient survival [36]. In contrast, intra-bone injection of MSC inhibited MM progression in preclinical settings [37]. Hence, there is a huge clinical need to unravel the multiple effects induced by tumor-MSC interactions and to demonstrate their impact on the course of disease.

In the present study, we investigated the expression of CCN1 in myeloma cells under conditions of a tumor-supportive microenvironment. We describe for the first time that myeloma cells display intron retention in *CCN1* mRNA, followed by splicing of all introns, enhanced transcription and consecutive translation upon contact with MSC. We also demonstrate that this effect can be exerted by recombinant CCN1 protein which further enhances myeloma cell viability.

## Results

### Contact with MSC induces *CCN1* pre-mRNA splicing, transcription, and CCN1 protein production in INA-6 multiple myeloma cells

**Figure 1:**
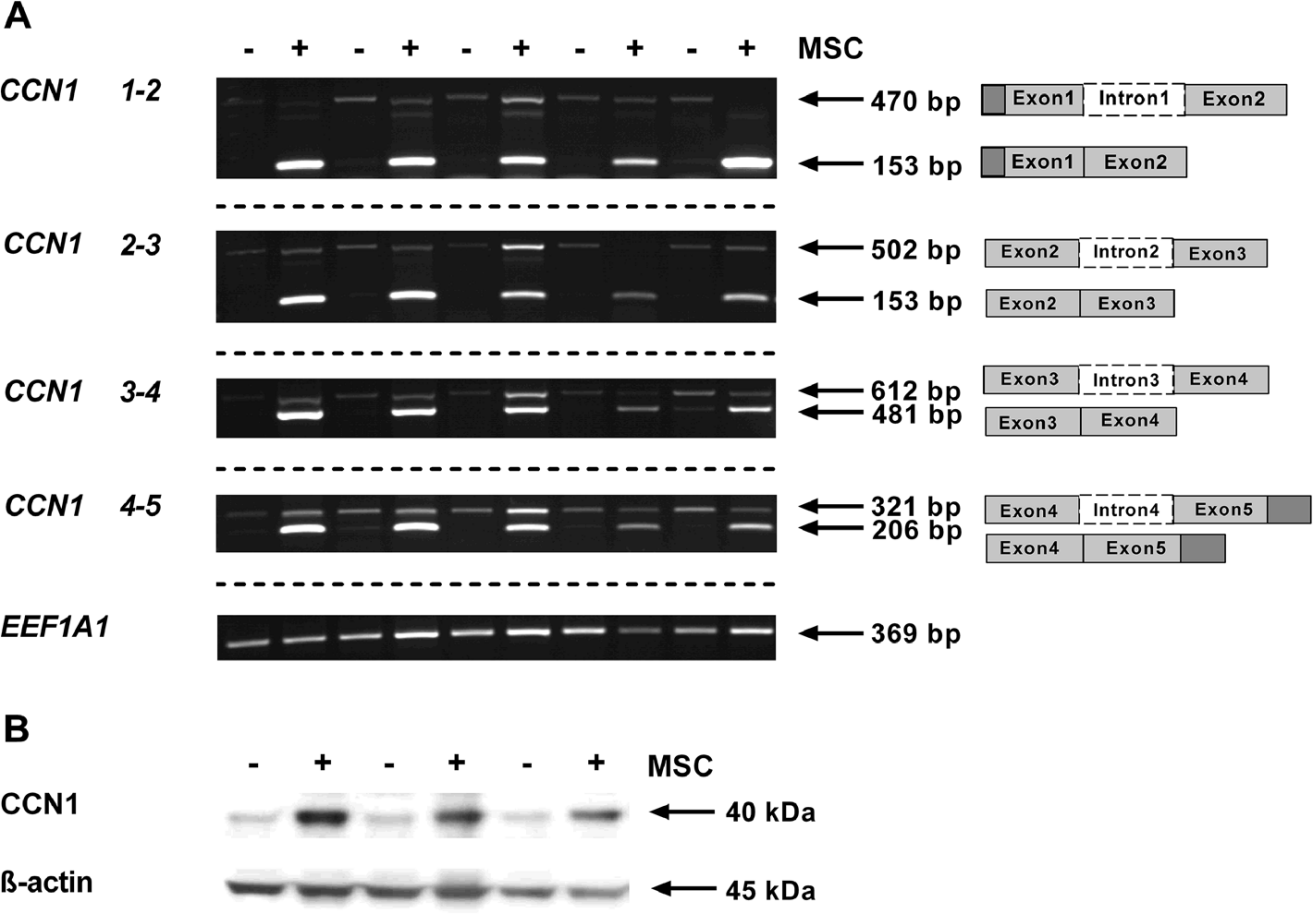
**Splicing of *CCN1 *pre-mRNA and protein expression is induced by MSC contact in INA-6 cells.** INA-6 cells were either cultured alone (-) or in the presence of MSC (+). After 24 h of co-culture, INA-6 cells were separated from MSC by washing. **(A)** Splicing pattern of *CCN1* pre-mRNA was investigated by semi-quantitative PCR with four different intron-spanning primers in five independent experiments. The primers are labeled according to their exon positions. INA-6 control cells (-) express the intron-retaining pre-mRNA whereas co-cultured INA-6 cells (+) show an increased expression of the intron-free transcript. Housekeeping gene *EEF1A1* served as control. Different gels are indicated by dashed lines. **(B)** CCN1 protein expression in INA-6 cells. Western blot analysis of three independent experiments shows an increased protein expression of CCN1 in INA-6 cells after MSC contact. Re-blotting for β-actin was used to confirm equal loading of protein.

To elucidate the role of CCN1 in the crosstalk between stromal cells and myeloma tumor cells, we performed co-culture experiments with primary MSC and the interleukin-6 (IL-6)-dependent myeloma cell line INA-6 [38]. Using intron-4 spanning primers (*CCN1 4*-*5*), INA-6 cells co-cultured with MSC expressed two alternate mRNA forms of different sizes (321 bp, 206 bp) compared to INA-6 control cells, which showed only a single variant (321 bp) (Figure 1A). Sequence analyses confirmed that the two variants represented the spliced (206 bp) and unspliced (321 bp) forms of *CCN1* mRNA. To evaluate if splicing is restricted to intron 4, additional primer pairs were designed to span the remaining three exon-intron transitions (Table 1). In each case, INA-6 control cells expressed the intron-retaining pre-mRNA, whereas INA-6 cells in contact with MSC expressed both the intron-retaining pre-mRNA and, to a greater extent, the intron-free isoform (Figure 1A). Overall, this pattern indicates that each intron of *CCN1* is affected by splicing upon contact with MSC. This was additionally confirmed by long-range PCR, as the combination of the 5′-primer *CCN 1*-*2* and the 3′-primer *CCN 4*-*5* showed two bands representing intron-retained and intron-free *CCN1* mRNA (data not shown). To elucidate if transcription of *CCN1* is enhanced in INA-6 cells after MSC contact, qPCR analyses were performed. *CCN1* mRNA expression in INA-6 cells co-cultured with MSC increased significantly by 5.3 fold (SD: 1.7-14.2: p < 0.001 calculated with REST [39]), with a high donor variability (2.4 to 17.3 fold), compared to control cells (data not shown). As intron-retaining *CCN1* sequence revealed a pre-mature stop codon in intron 1 (Additional file 1: Figure S1), translation of full-length CCN1 protein, as well as truncated isoforms, could be excluded in INA-6 control cells since exon 1 only encodes for the signal peptide, whereas intron-free *CCN1* mRNA expressed by INA-6 cells after MSC contact should be able to translate the full-length protein. To analyze protein expression of CCN1, INA-6 control cells, as well as MSC co-cultured INA-6 cells, were investigated by western blot using the polyclonal antibody CYR61 H-78, raised against amino acids located in the “hinge-region”. Western blot analyses demonstrated that INA-6 cells co-cultured with MSC showed a 345% increase in CCN1 protein levels in contrast to INA-6 control cells cultured alone (Figure 1B). As only a small number of MSC reside within the bone marrow, we additionally performed co-culture experiments with INA-6 and osteoblasts, generated by incubating MSC with osteogenic differentiation media for 14 days. *CCN1 4*-*5* primers confirmed a similar splicing pattern in INA-6 cells obtained after co-culture with MSC (data not shown).

**Table 1:** **Primer sequences and conditions of semi**-**quantitative PCR**

**Gene product**	**Primer 5**′-**3**′ **sequence**		**Annealing temperature (°C)**	**Length of PCR product (bp)**	**mRNA isoform**
*CCN1 1*-*2*	Reverse	TCACCCTTCTCCACTTGACC	56	470	Intron-retained
	Forward	AGTCCTCGTTGAGCTGCTTG		153	Intron-free
*CCN1 2*-*3*	Reverse	ACCGCTCTGAAGGGGATCT	56	502	Intron-retained
	Forward	GGGACACAGAGGAATGCAG		153	Intron-free
*CCN1 3*-*4*	Reverse	GGCAGACCCTGTGAATATAA	50	612	Intron-retained
	Forward	CAGGGTTGTCATTGGTAACT		481	Intron-free
*CCN1 4*-*5*	Reverse	CAACCCTTTACAAGGCCAGA	54	321	Intron-retained
	Forward	TGGTCTTGCTGCATTTCTTG		206	Intron-free
*EEF1A1*	Reverse	CTGTATTGGATTGCCACG	54	369	
	Forward	AGACCGTTCTTCCACCACTG			

### Exogenous stimulation by recombinant CCN1-Fc protein induces *CCN1* pre-mRNA splicing and transcription in INA-6 cells in a concentration-dependent manner

**Figure 2:**
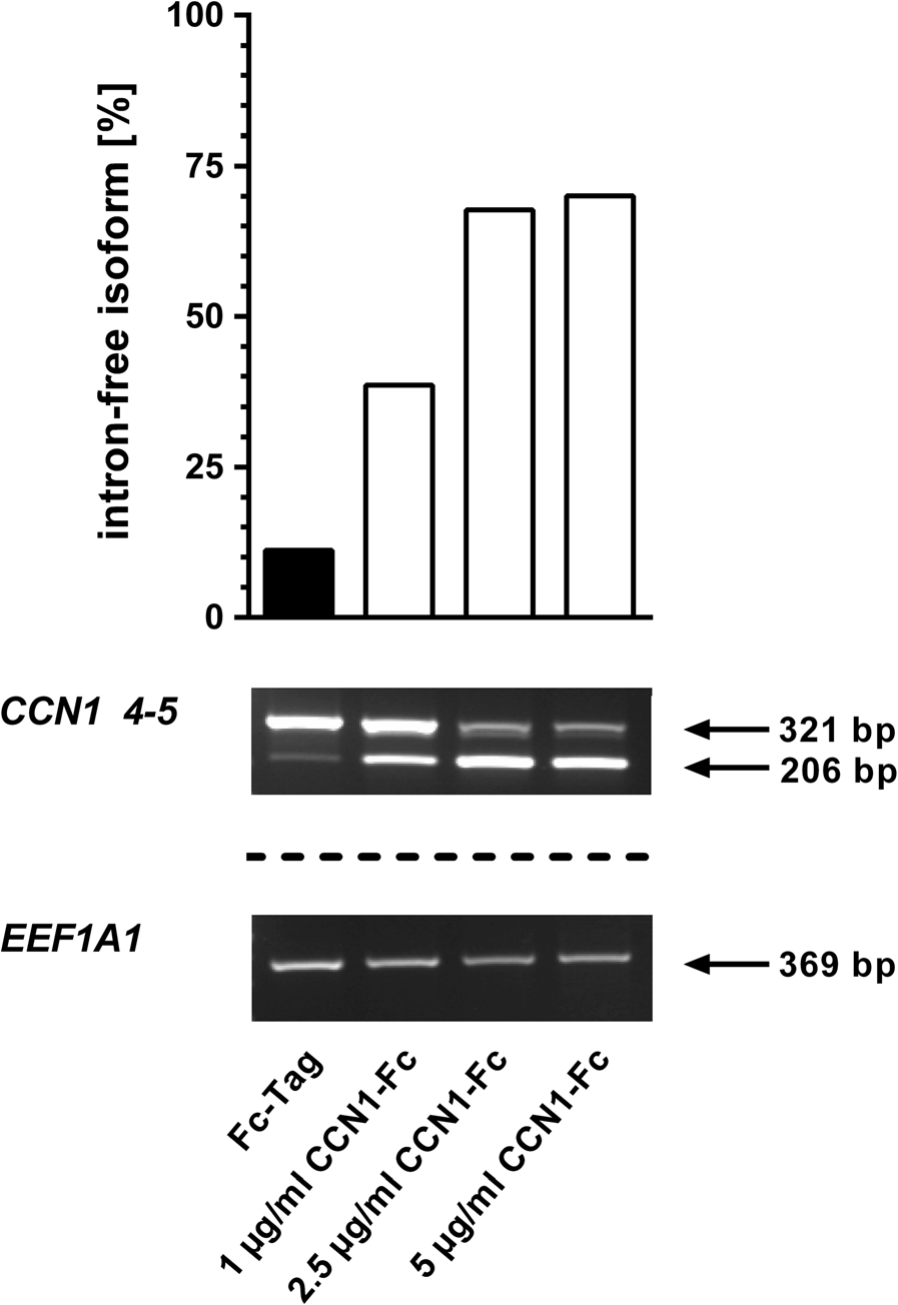
**Exogenous stimulation by recombinant CCN1-Fc induces splicing of *CCN1 *pre-mRNA in INA-6 cells.** INA-6 cells were incubated with varying concentrations of CCN1-Fc for 24 h. Semi-quantitative PCR was performed with primers for *CCN1 4*-*5* and *EEF1A1*. Densitometric analysis was used to determine the amount of intron-retaining and intron-free pre-mRNA isoform. Bar graphs showing the percentage of intron-free isoform (intron-free mRNA compared to total (intron-free + intron retained) mRNA), which is enhanced by increased concentration of CCN1-Fc. *EEF1A1* was used to normalize data.

Hirschfeld *et al*. previously described the splicing of intron 3 in *CCN1* pre-mRNA in association with breast cancer progression as an oxygen- and pH-regulated event [17,18]. In our case, neither incubation in hypoxic (1% O_2_) nor acidic conditions led to splicing of *CCN1* in INA-6 cells (data not shown). Furthermore, indirect co-culture experiments with trans-well inserts (data not shown), as well as experiments with MSC-derived conditioned media (MSC-CM) (Additional file 2: Figure S2), was not sufficient to induce *CCN1* mRNA splicing in INA-6 cells. It has previously been shown that exogenous stimulation using recombinant CCN1 protein induced endogenous CCN1 production [40]. To determine if this effect was mediated through splicing, we investigated if splicing of *CCN1* pre-mRNA occurs after treatment with recombinant protein. Although CCN1 is highly expressed by MSC [8], native protein concentration may rapidly diminish in solution due to aggregation on MSC cell surface or cell culture dishes. Therefore, exogenous stimulation of INA-6 cells was performed by using an Fc-tagged recombinant CCN1 protein (CCN1-Fc), which is more stable in solution. INA-6 cells were incubated with varying concentrations of recombinant CCN1-Fc protein for 24 h and analyzed by PCR with *CCN1* intron-4 spanning primers (*CCN1 4*-*5*). Results show that treatment with CCN1-Fc induced splicing of *CCN1* pre-mRNA in a concentration-dependent manner, as represented by an increased amount of intron-free RNA isoform compared to total *CCN1* mRNA (Figure 2). Incubation with 1 μg/ml CCN1-Fc increased the percent of *CCN1* mRNA in its intron-free form from 10%, observed before recombinant protein addition, to 40% after protein addition. This increased prevalence of intron-free *CCN1* mRNA was further enhanced to ~ 70% intron-free mRNA by incubation with 2.5 μg/ml of recombinant protein. To evaluate if splicing is not only concentration dependent, but also time dependent, we performed time course experiments after 4 h, 8 h and 24 h. Concentrations of CCN1-Fc which showed small to medium splicing activity after 24 h incubation were used to allow detection of enhanced splicing. Splicing plateaued within 4 h of incubation with recombinant protein concentrations as low as 0.05 μg/ml (Additional file 3: Figure S3), and remained consistent for up to 72 h after stimulation with CCN1-Fc (data not shown). Splicing of *CCN1* pre-mRNA appears to be a rapid, concentration-dependent event in response to CCN1 protein stimulation. To determine changes at the transcription level of *CCN1* mRNA in response to CCN1-Fc treatment, we measured overall intensity of bands obtained with intron-4 spanning primers by semi-quantitative PCR and densitometric analyses. Relative to mRNA expression in Fc-Tag treated control cells, incubation with 1 μg/ml or 5 μg/ml CCN1-Fc led to a 1.8 fold increased expression of *CCN1* (Additional file 4: Figure S4A). Similar results were obtained by qPCR with a second intron-4 spanning primer pair. Treatment with 1 μg/ml CCN1-Fc enhanced *CCN1* mRNA expression by 1.4 fold, 2.5 μg/ml CCN1-Fc led to a 2.5 fold enhanced gene expression, which diminished to 2.1 fold after stimulation with 5 μg/ml CCN1-Fc in comparison to Fc-Tag control (Additional file 4: Figure S4B).

### Domain 4 of CCN1-Fc is involved in inducing *CCN1* pre-mRNA splicing

**Figure 3:**
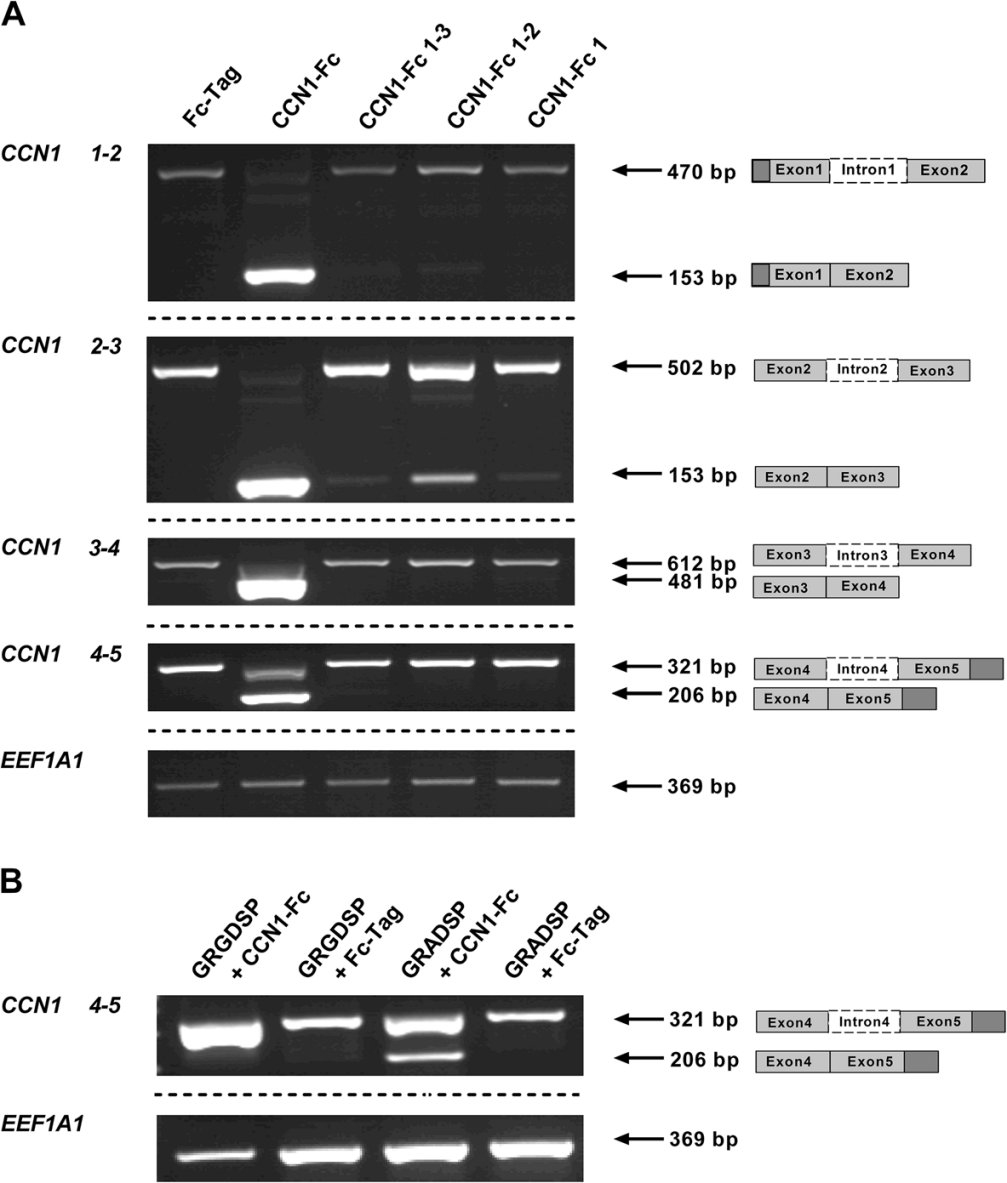
**Domain 4 of CCN1-Fc is required to induce *CCN1 *pre-mRNA splicing. (A)** INA-6 cells were incubated with either 2.5 μg/ml recombinant CCN1-Fc full-length protein, or an equivalent amount of truncated CCN1 isoforms (CCN1-Fc 1-3, CCN1-Fc 1-2, and CCN1-Fc 1). Semi-quantitative PCR with intron-containing *CCN1* primers showed that exposure to truncated CCN1 proteins comprising domains 1, 1-2, or 1-3 did not induce complete *CCN1* mRNA splicing. **(B)** Splicing of *CCN1* pre-mRNA induced by 2.5 μg/ml full-length CCN1-Fc was reversed by GRGDSP peptide (50 μg/ml in culture media) but not by addition of the control peptide GRADSP. One representative experiment out of three is shown.

To characterize which domains of CCN1 are involved in stimulating mRNA intron splicing, we incubated INA-6 cells with full length CCN1-Fc, as well as truncated isoforms comprising domain 1 (CCN1-Fc 1), domains 1 and 2 (CCN1-Fc 1-2) or domains 1-3 (CCN1-Fc 1-3). Only the full length CCN1-Fc was able to induce the splicing of *CCN1* mRNA for all introns while induction with truncated CCN1-Fc showed only limited splicing of introns 1 and 2 (Figure 3A), highly suggestive that domain 4, also known as the cysteine-knot containing module (CT), is necessary for the induction of splicing of all introns. Because CCN protein functions are mediated mainly by integrin binding [41] and because domain 4 contains several integrin binding sites, we next blocked integrin-binding activity on INA-6 cells by pre-incubation with GRGDSP peptide, followed by the addition of full length CCN1-Fc. Incubation with GRGDSP peptide inhibited splicing of intron 4 in contrast to the control peptide GRADSP, which failed to inhibit splicing of intron-free mRNA (Figure 3B). Comparing integrins that are able to bind RGD motif in their ligands [42] with integrin-binding sites located in the last domain of CCN1 [10], only *α*_*v*_*β*_*3*_ integrin matched. To determine the type of integrin responsible for the induction of *CCN1* pre-mRNA splicing, INA-6 cells were incubated with *α*_*v*_*β*_*3*_ blocking antibody and CCN1-Fc, which showed no reduction in splicing (data not shown). We further performed blocking experiments with anti-integrin *β*_*1*_, *β*_*2*_, *β*_*3*_, *β*_*7*_ antibodies, as these integrins are described as being expressed on myeloma cells [43], and integrins *α*_*6*_*β*_*1*_ and *α*_*M*_*β*_*2*_, which interact with the CT-domain of CCN1 [10]. No anti-integrin antibody was able to block splicing mediated by stimulation with CCN1-Fc (data not shown).

### CCN1-Fc enhances INA-6 cell viability in the absence of IL-6

**Figure 4:**
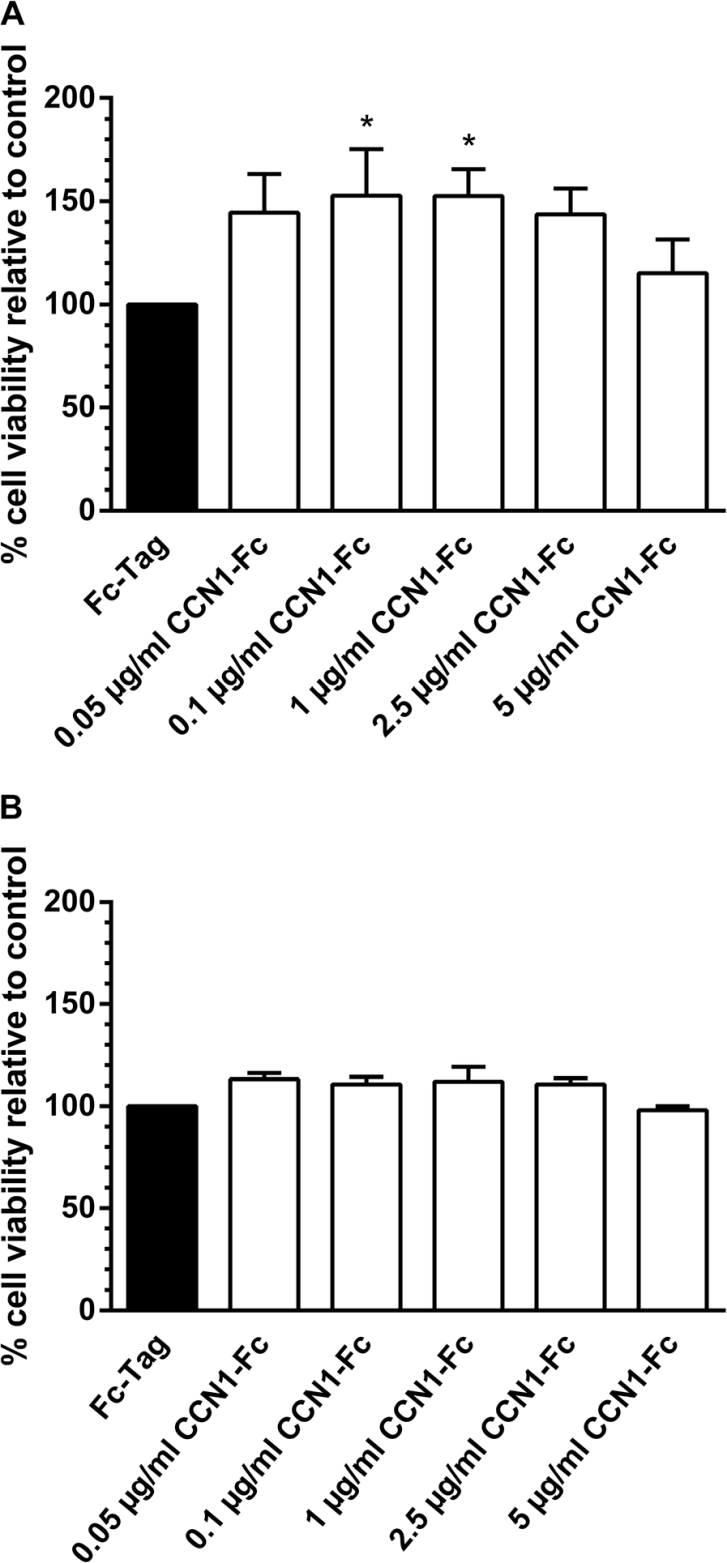
**CCN1-Fc enhances INA-6 cell viability in the absence of IL-6.** INA-6 cells were cultured with serum-reduced media for 24 h before stimulation with **(A)** CCN1-Fc or **(B)** CCN1-Fc and IL-6. After 24 h, cell viability was measured by quantification of ATP. In the absence of IL-6, INA-6 cell viability was significantly enhanced when stimulated with 0.1 μg/ml – 1 μg/ml CCN1-Fc, whereas incubation with 0.05 μg/ml – 2.5 μg/ml CCN1-Fc led to a slight enhancement of INA-6 cell viability in presence of IL-6. Results depicted graphically represent the mean + SD for four independent experiments. *p < 0.05 relative to control.

It has previously been shown that survival of both INA-6 cells and primary myeloma cells is independent of IL-6 when cultured with MSC [38]. We next wanted to investigate whether CCN1, as a MSC-derived factor, has a positive effect on myeloma cell viability. Therefore, pre-starved INA-6 cells were incubated with varying concentrations of CCN1-Fc for 24 h, in the absence of IL-6, and cell viability was assessed through quantification of ATP content. Cell viability was increased by ~40% when treated with 0.05 μg/ml or 2.5 μg/ml CCN1-Fc and further significantly enhanced to 50% when cells were incubated with 0.1 μg/ml or 1 μg/ml CCN1-Fc compared to control cells treated with Fc-Tag (Figure 4A). Enhanced cell viability was not accompanied with reduced apoptosis induction or enhanced phosphorylation of STAT3, Erk1/2 or p38 MAPK (data not shown). Additionally, in the presence of IL-6, incubation with 0.05 μg/ml - 2.5 µg/ml CCN1-Fc showed only a marginal 15% increase in cell viability while treatment with 5 μg/ml CCN1-Fc had no effect (Figure 4B).

## Discussion

In the present study, we expand knowledge on regulatory mechanisms involving splicing of the proangiogenic, matricellular growth and differentiation factor CCN1/CYR61 in the tumor microenvironment with a special focus on myeloma-MSC interaction. Our new findings include mechanistic data on the role of intron retention and splicing as a means of translation regulation of immediate early proteins of the CCN family as well as putative biological functions of CCN1 as a factor promoting myeloma cell viability.

CCN1, according to genetic mouse models and *in vitro* signaling experiments, is involved in angiogenesis and anoikis [4,6,14,44,45]. It is expressed in regenerating/ proliferating MSC and osteoblast precursors but not in mature bone cells [7,8]. Earlier and recent reports have demonstrated that CCN1 is expressed in MM myeloid and lymphoid cells, as well as other tumor cells such as breast and prostate cancer, and may contribute to tumor cell survival by providing signals stimulating angiogenesis and survival through acquire anoikis resistance [14,44,46-48]. We observed *CCN1* intron splicing in INA-6 myeloma cells for all four introns of the *CCN1* mRNA and enhanced transcription of *CCN1* after co-culture with MSC. This extends previous findings of intron splicing with regard only to intron 3 in *CCN1* mRNA of breast cancer cells [18]. The presence of 2 pre-mature stop codons within this intron 3-retaining mRNA sequence suggested the possible existence of truncated CCN1 proteins containing domain 1 and 2. However, our finding of intron 1-4 retention in myeloma cells, and the presence of an in-frame stop codon downstream to the *CCN1* signal sequence (within intron 1) implies that intron 1-4 retaining *CCN1* mRNA completely blocks endogenous translation of full length CCN1 protein in INA-6 cells, preventing translation of truncated variants that could be termed isoforms resulting from alternative splicing [15]. Moreover induction of splicing through contact with MSC is initiated at all introns and allows for full length protein expression in INA-6 cells, as confirmed by the increase in CCN1 protein using an antibody directed towards the hinge region. As this part of the protein offers the most diversity to the other members of the CCN family [11], antibodies directed against the variable linker region will cause less cross-reactivity to other CCN proteins and higher specificity than domain-specific antibodies.

In the case of breast cancer, alternative splicing of intron 3 in *CCN1* pre-mRNA is regulated by hypoxia and acidosis which further leads to disease progression [17,18]. In the case of MM, changes in *CCN1* mRNA splicing patterns appear independent of pH change and oxygen deficiency, nor is it mediated by soluble factors, as indirect co-culture with MSC using trans-well inserts, as well as by incubating with MSC-derived conditioned media, was not sufficient to induce *CCN1* splicing in INA-6 cells. However, recombinant human Fc-tagged CCN1 protein was capable of inducing this phenomenon. We therefore hypothesized that the native CCN1 protein is rapidly aggregating on cell surfaces and materials of cell culture flasks, and thus, is not available (in sufficient quantity) in the soluble phase. This hypothesis is further confirmed by previous findings that CCN1, although it contains an N-terminal secretory signal peptide, is not secreted into the supernatant and instead, remains associated with the extracellular matrix, leading to restriction of CCN1 action to the local microenvironment [49]. In contrast the recombinant CCN1-Fc protein shows greater stability in solution and induces *CCN1* mRNA splicing when added to cell cultures. As the amount of intron-free isoform of *CCN1* mRNA increased compared to total *CCN1* mRNA, and because increases in the intron-free isoform is accompanied with a decrease in the intron-retained isoform, we suspect that splicing induction is an important regulatory mechanism. In addition, densitometric analyses and qPCR revealed that incubation with CCN1-Fc further led to enhanced transcription of *CCN1* mRNA, and that this enhancement is a rapid process which responds in a dose-dependent manner. As well as being a rapid process, concentrations as low as 0.05 μg/ml CCN1-Fc was sufficient to induce splicing, suggesting a highly sensitive response to CCN1 levels. Additionally, once primed with CCN1-Fc, splicing patterns were detectable up to three days after stimulation, suggesting a long-lasting signature. We conclude that CCN1 protein, delivered in a paracrine manner, is an appropriate stimulus to initiate complete splicing of intron-retaining *CCN1* mRNA in myeloma cells, which further leads to protein translation in the target cell, maintaining further CCN1 expression in an autocrine manner. The fact that CCN1 production in histological sections of MM patients is located in cells that are not directly located adjacent to bone-derived cells favors a long term signature in MM cells, which remains after contact with CCN1-producing bone cells, though other stimulators cannot be excluded. Production of CCN1 by myeloma cells in addition to CCN1 derived from cells in the bone marrow may further lead to an angiogenic switch that is associated with disease progression [50].

CCN proteins are involved in many different cellular functions depending on their interacting partners - mainly integrins - expressed on the cell surface. The CT domain, as well as the TSR and vWC domain, include several integrin-binding sites that promote the activity of CCN proteins [5,11,51]. The CT domain contains several integrin-binding sites for *α*_*6*_*β*_*1*_, *α*_*M*_*β*_*2*_, and *α*_*v*_*β*_*3*_ integrins, whereas the integrin *α*_*6*_*β*_*1*_ can bind the TSR domain and integrin-binding sites for *α*_*v*_*β*_*3*_, *α*_*v*_*β*_*5*_, and *α*_*IIb*_*β*_*3*_ are located within the vWC domain [10,51]. Several integrins, such as *α*_*v*_*β*_*3*_ and *α*_*v*_*β*_*5*_, bind their ligands by recognizing RGD sequences [42]. To elucidate the mechanism involved in splicing, we incubated INA-6 cells with full length and truncated CCN1 proteins that contain domain 1-3, 1-2, or domain 1 only. Polypeptides lacking domain 4 did not show any effect, suggesting that domain 4, also known as the CT domain, is necessary for splicing induction. Furthermore, blocking RGD-dependent integrins on INA-6 cells by pre-incubation with GRGDSP peptide abolished CCN1-induced splicing in INA-6 cells, supporting the hypothesis of an integrin-mediated mechanism. CCN1 does not contain an RGD sequence motif, but RGD-blocking peptides have been previously reported to also abolish other effects of CCN proteins [41]. Blocking antibodies targeting β-integrins (*β*_*1*_, *β*_*2*_, *β*_*3*_, and *β*_*7*_) that are relevant in myeloma [43], as well as *α*_*v*_*β*_*3*_, the RGD-sensitive integrin comprised in CT domain, were unable to prevent the splicing induction of *CCN1* mRNA by CCN1-Fc stimulation. We can only speculate on the reason for this discrepancy, one of which may be that binding sites involved are not targeted by hitherto available antibodies. Thus, we suggest that splicing induction may be a complex mechanism, dependent on engagement of several integrins and/or co-receptors that require further investigations.

In general, multiple myeloma cells, if removed from the bone marrow, often cannot survive without feeder cells or supplementation of factors to cell cultures, e.g. IL-6 in case of the INA-6 cell line [52]. In the case of co-culture with MSC, myeloma cells survive independent of the IL-6/gp130/STAT3 pathway [38] suggesting that other pro-myeloma factors, such as CCN1, are provided by the stromal tumor microenvironment. We were able to detect a significantly increased cell viability of myeloma cells after CCN1 incubation independent of IL-6 suggesting CCN1 as a microenvironment-derived pro-myeloma factor. CCN1-dependent signaling pathways were not involved mechanistically in this pro-myeloma effect as no alterations in phosphorylated p38, ERK1/2, and STAT3 by CCN1 treatment of INA-6 cells was observed.

## Conclusions

Taken together, we show that INA-6 myeloma cells are able to produce functional CCN1 protein after MSC contact, which is induced by a splicing event concerning all introns as well as enhanced transcription. MSC-dependent splicing that governs CCN1 protein translation, as shown here, is extremely relevant in both physiologic processes like regeneration and in tumor development, e.g. by enhancing angiogenesis, especially in myeloma bone disease. MSC have been reported to support or to suppress tumorigenesis and this is certainly one of the more protective effects [53]. MSC-based treatment strategies in MM must be critically examined with regard to the oncogenic splicing switch potential of CCN1. In order to survive within the bone marrow, MM cells produce a series of modulatory factors for osteogenic differentiation to suppress e.g. osteoblast-derived inhibitory factors for MM growth and survival [26,32,54,55]. In addition, MM cells, by either physical contact or humoral factors, stimulate stromal osteogenic precursors to produce supportive factors, e.g. IL-6. Such contact-mediated signatures of the transcriptome in stromal osteogenic precursors may also persist as a permissive situation in myeloma cells. Our findings suggest CCN1 as a MSC-induced factor with relevance in tumor relapse and development of secondary malignancies.

## Methods

### Ethics statement

Bone material was used according to the permission of the local Ethics Committee (Medical Faculty of the University of Wuerzburg) and after having obtained written, informed consent of each patient.

### Reagents and antibodies

Primary antibodies for western blot analyses were directed against: CCN1 (CYR61 H-78, rabbit, sc-13100; Santa Cruz Biotechnology, Heidelberg, Germany), Tyr705-phosphorylated STAT3 (rabbit mAb, #9145; New England Biolabs (NEB), Beverly, MA, USA), Thr202/Tyr204 phosphorylated p44/42 MAPK (ERK1/2) (rabbit, #9101; NEB), and Thr180/Tyr182 p38 MAPK (rabbit mAb, #9215; NEB). Loading control antibodies were specific for: β-actin (rabbit mAb, #4970; NEB), STAT3 (rabbit, #4904; NEB), p42/44 MAPK (mouse mAb, #4696; NEB), and p38α MAPK (mouse mAb, #9217; NEB). Anti-rabbit antibody (goat-anti-rabbit IgG (whole molecule)-peroxidase, A0545; Sigma Aldrich Chemie (Sigma), Schnelldorf, Germany) and anti-mouse antibody (goat-anti-mouse IgG (Fab specific)-peroxidase, A9917; Sigma) were used as secondary antibodies. To block RGD-dependent integrins, blocking peptide (Gly-Arg-Gly-Asp-Ser-Pro, GRGDSP, SCP0157) and control peptide (Gly-Arg-Ala-Asp-Ser-Pro, GRADSP, SCP0156) were purchased from Sigma. The following functional-blocking antibodies against several integrins were obtained: β1 (monoclonal mouse IgG1, MAB 17781; R & D Systems (R & D), Wiesbaden, Germany), β2 (antigen affinity purified polyclonal goat IgG, AF 1730; R & D), β3 (monoclonal mouse IgG1, NBP1-28398; Novus Biologicals, Littleton, CO, USA), β7 (monoclonal rat IgG2a, κ, 321218; BioLegend, San Diego, CA, USA), *α*_*v*_*β*_*3*_ (monoclonal mouse IgG1, MAB 3050; R & D). Isotype antibodies were used as control: Rat IgG2a, κ (400516; BioLegend), mouse IgG1 (MAB002; R & D), normal goat IgG (sc-2028; Santa Cruz).

### Cell culture

Primary MSC of healthy donors were isolated from bone marrow of femoral heads after total hip arthroplasty and characterized as previously described [8,56]. MSC were selected by surface adherence and expanded in DMEM/Ham’s F-12 (1:1) (PAA Laboratories (PAA), Linz, Austria) supplemented with 10% (vol/vol) FCS (Biochrom, Berlin, Germany), 1 U/ml penicillin, 100 μg/ml streptomycin (PAA) and 50 μg/ml L-ascorbic acid 2-phosphate (Sigma). All experiments were performed with MSC in passage 1. Osteogenic precursor cells (OPC) were generated by incubating MSC with osteogenic differentiation media for 14 days. Osteogenic differentiation media consisting of DMEM High Glucose media (PAA) with 10% (vol/vol) FCS, 1 U/ml penicillin, 100 μg/ml streptomycin and additionally supplemented with 10 mM β-glycerophosphate disodium salt hydrate (Sigma), 100 nM dexamethasone (Sigma) and 50 μg/ml L-Ascorbic acid-2-phosphate sesquimagnesium salt hydrate (Sigma). Osteogenic differentiation was checked with histological staining for alkaline phosphatase using the Alkaline Phosphatase, Leukocyte Kit 86-C (Sigma) and mineralized extracellular matrix was visualized by staining with alizarin red S (Sigma). The cell line INA-6 [52] was maintained in RPMI 1640 complete medium that is generated by adding 20% (vol/vol) FCS, 100 μg/ml gentamycine, 2 mmol/l glutamine (PAA), 1 mmol/l Na-pyruvate (Sigma) to RPMI 1640 media (PAA, Linz, Austria). INA-6 cells were cultivated with a final concentration of 2 ng/ml recombinant human interleukin-6 (IL-6; R & D). For measuring cell viability and apoptosis, INA-6 cells were pre-starved by incubating with serum reduced RPMI 1640 complete medium containing 4% (vol/vol) FCS and 2 ng/ml IL-6 for 24 h. Starvation media permits cultivation of INA-6 cells over 48 h without inducing enhanced cell death compared to control (proved by trypan blue exclusion). All cell cultures were regularly tested for mycoplasma and grown at 37°C and 5% CO_2_. For experiments under hypoxic conditions, cultures were incubated in an incubator adjusted to 1% O_2_. In parallel, control cells were cultured under normoxia (21% O_2_). Acidic conditions were generated by culturing INA-6 cells in consumed media.

### Direct and indirect co-cultures of MM cells with MSC

5×10^5^ MSC were seeded per well (6-well plate) and given time to attach for one day. 2×10^6^ INA-6 cells, washed once with PBS, were either added to the MSC or cultured with 2 ng/ml IL-6 (control) in a mixture of DMEM/Ham’s F12 and RPMI 1640 (1:1). After 24 h of co-culture, INA-6 cells were carefully rinsed off the MSC layer with PBS. To prevent contamination of INA-6 cells with MSC, rinsing was performed with regard to leaving the MSC layer intact as well as by staining INA-6 cells with CellTracker™ Green 5-chloromethylfluorescein diacetate (CMFDA, 5 μM; PA-3011, Lonza Group, Basel, Switzerland) according to the manufacturer’s instructions before performing co-culture. Detached INA-6 cells were examined afterwards by using a fluorescence microscope (Microscope Axiovert 25; Zeiss, Jena, Germany). As no unstained cells were detected, contamination with MSC was excluded. In the case of indirect co-culture using trans-well inserts, MSC were seeded as previously described on 6-well tissue culture polystyrene plates (Corning, NY, USA) and INA-6 cells were transferred on polyester membrane insert (0.4 μm pore size).

### Preparation of MSC-derived conditioned media (MSC-CM)

MSC-CM was generated from MSC supernatant after 48-72 h of culture after passing through a 0.2 μm filter to eliminate remained cellular debris and contaminating cells. In parallel, 2×10^6^ INA-6 cells were centrifuged at 1200 rpm and cell pellet was resuspended in 5 ml MSC-CM or, in the case of control, in 5 ml MSC media, supplemented with 2 ng/ml IL-6 and incubated for 24 h. The next day, cells were harvested for RNA isolation.

### Protein extraction and western blot analyses

For CCN1 detection, INA-6 control cells were cultured with 2 ng/ml IL-6 or co-cultured with MSC and harvested as described above. To investigate signaling pathways, INA-6 cells were incubated with IL-6-deprived media for 20 h, washed, and stimulated with CCN1-Fc or Fc-Tag for 30 min or 60 min after starving for 4 h in serum-free media.

To generate whole cell lysates, cells were washed with PBS and incubated with lysis buffer (#9803; NEB) supplemented with complete, EDTA-free protease inhibitor cocktail (#04693132001; Roche, Mannheim, Germany) and PhosStop phosphatase inhibitor cocktail (#0496845001; Roche) for 10 min on ice. After sonication with an ultrasonic homogenizer (Sonopuls, Bandelin), cell lysates were cleared by centrifugation (12000 rpm, 15 min, 4°C). Protein concentrations were determined using the Bradford assay (Roti®-Quant; Carl Roth, Karlsruhe, Germany). Equal amounts of proteins mixed with Laemmli buffer were heated up to 95°C for 5 min and run on sodium dodecyl sulphate 10%-polyacrylamide gels before blotting on polyvinyldifluoride (PVDF) membranes (WESTRAN® clear signal; GE Healthcare Europe GmbH, Freiburg, Germany). Blots were incubated in blocking buffer (p38α MAPKp44/42 MAPK (Erk1/2), After four washes with buffer, the blots were incubated with corresponding peroxidase-conjugated secondary antibody in corresponding blocking buffer for 2 h at room temperature. Visualization of the blots was performed with the enhanced chemiluminescence (ECL) system (Amersham ECL Prime Western Blotting Detection Kit; GE Healthcare Europe GmbH, Freiburg, Germany) and X-ray films (Fuji Medical X-ray film Super RX, Fujifilm Europe GmbH, Düsseldorf, Germany). Loading was determined by stripping the membrane and re-blotting with corresponding control antibody.

### RNA isolation and polymerase chain reaction (PCR)

Total RNA was isolated from INA-6 cells using the NucleoSpin® RNA II kit (Machery-Nagel GmbH & Co. KG, Düren, Germany) according to the manufacturer’s instructions. Purity and yield of the RNA were photometrically determined. cDNA was synthesized by using 1 μg of total RNA, random primers (Life Technologies GmbH, Darmstadt, Germany), and M-MLV reverse transcriptase (Promega GmbH (Promega), Mannheim, Germany) according to the manufacturer’s instructions.

Semi-quantitative PCR analyses were carried out in a volume of 30 μl containing 6 μl 5× Green GoTaq® Flexi Buffer (#M891A, Promega), 20 ng cDNA, 1.7 mM MgCl_2_ (#A351H; Promega), 0.3 mM dNTPs (Bioline, Luckenwalde, Germany), 1 unit GoTaq® DNA Polymerase (Promega) and 5 pmol sequence specific primers (Eurofins MWG Operon, Ebersberg, Germany) for *CCN1* gene or the housekeeping gene *eukaryotic translation elongation factor 1 alpha 1* (*EEF1A1*). Intron-spanning primer sequences for *CCN1* (accession number DNA sequence: NC_000001.10) were obtained by using the online software Primer3Plus (see Table 1 for primer sequences). PCR was run in a peqSTAR 2× thermocycler (Peqlab Biotechnologie GmbH, Erlangen, Germany) as follows: 2 min at 95°C, 95°C for 30 sec, 50-56°C for 30 sec (*EEF1A1* and intron-4 spanning *CCN1* primer (*CCN1 4*-*5*)) or 45 sec, and 72°C for 1 min (37-43 cycles for *CCN1*, 22-25 cycles for *EEF1A1*), finished by 72°C for 3 min. For long-range PCR primer pairs *CCN1 1*-*2* forward and *CCN1 4*-*5* reverse were used. Annealing temperature was adjusted to 52°C, elongation time to 2 min. Separation of 10 μl of PCR products occurred on a 2% agarose gel containing GelRed® (#M3199; Genaxxon Bioscience GmbH, Ulm, Germany). Specificity of PCR products was verified by sequence analyses using Big Dye Terminator v1.1 Cycle Sequencing Kit and ABI 3130xL Genetic Analyzer (Applied Biosystems, Darmstadt, Germany).

Quantitative real-time PCR (qPCR) was performed in a volume of 20 μl with 1 μl of cDNA, which was previously diluted 1:5, 10 μl KAPA SYBR FAST Universal qPCR Master Mix (Peqlab Biotechnologie GmbH, Erlangen, Germany) and 1 pmol each forward and reverse primers (biomers.net GmbH, Ulm, Germany) by using Opticon DNA Engine (MJ Research, Watham, USA). Amplification of *CCN1* gene was performed by using primers specific for exons 4-5 (5′-primer, 5′-ACGAGTTACCAATGACAACC-3′; 3′-primer, 5′-CCAGCGTAAGTAAACCTGAC-3′). As an internal control, mRNA expression level of housekeeping gene *RPS27A* was amplified with following sequence specific primers (5′-primer; 5′-TCGTGGTGGTGCTAAGAAAA-3′; 3′-primer, 5′-TCTCGACGAAGGCGACTAAT-3′). QPCR conditions were as follows: 95°C, 3 min; 40 cycles: 95°C, 15 s; 60°C for RPS27A, 57°C for *CCN1*, 15 s; 72°C, 20 s; followed by melting curve analysis for specificity of qPCR products. qPCRs were performed three times, results were calculated with the ΔΔct method and significances with the Relative Expression Software Tool (REST 2009 V2.0.13) obtained from Qiagen GmbH (Hilden, Germany) [39].

### Expression and purification of recombinant CCN1 protein (CCN1-Fc) and CCN1 domains (CCN1-Fc 1-3, CCN1-Fc 1-2, CCN1-Fc 1)

The cloning of *CCN1*, as well as the preparation of the virus stock of CCN1-Fc, was generated as previously described [57]. Additionally, to express recombinant CCN1-Fc proteins lacking domain 4 (CCN1-Fc 1-3), domains 3 and 4 (CCN1-Fc 1-2) or domains 2-4 (CCN1-Fc 1), respectively, corresponding open reading frames were similarly cloned and expressed. To purify the CCN1-Fc protein, as well as the domain-lacking proteins or Fc-Tag protein, 1 ml HiTrap Protein G HP columns were used with a peristaltic pump P-1 (GE Healthcare Europe GmbH, Freiburg, Germany). 75 ml cell supernatant was filtered (0.2 μm) and transferred to a PBS equilibrated column. Columns were washed with PBS for 15 min before protein was eluted with elution buffer (0.1 M glycine, pH 2.2). Previously provided 3M Tris/HCl, pH 8 immediately neutralized eluted protein fractions. Protein was further used for gel electrophoresis and purity was checked by silver staining and western blotting using Fc-Tag antibody, as previously described [57]. Proteins were stored at -20°C until use. After thawing, the tube was centrifuged and after transferring the supernatant into a fresh tube, protein concentration was determined by Bradford assay.

### Treatment of INA-6 cells with CYR61-Fc and truncated CYR61-Fc proteins

INA-6 cells were seeded in 24 well-plates (3×10^5^ cells/well) and treated with CCN1-Fc, truncated CCN1-Fc isoforms, or Fc-Tag for control. The quantity of truncated proteins or Fc-Tag was added in equimolar concentration compared to the full-length protein CCN1-Fc. At indicated time points, cells were harvested for RNA isolation.

For blocking integrin signaling, INA-6 cells were seeded as mentioned above and pre-incubated for 45 min with 55 μg/ml GRGDSP peptide (control: GRADSP peptide) or 22 μg/ml anti-integrin antibody (control: isotype antibody) at 37°C and 5% CO_2_. For 24 h incubation, CCN1-Fc or Fc-Tag was added as previously described at a final concentration of 50 μg/ml peptide or 20 μg/ml anti-integrin antibody.

### Cell viability assays

Pre-starved INA-6 cells were washed once with PBS, resuspended in starvation media with or without IL-6 and seeded in a 96-well plate (1250 cells/well). To assess the influence of CCN1-Fc on cell viability, INA-6 cells were stimulated in triplicate for 24 h with CCN1-Fc at a final concentration ranging from 0.1 μg/ml to 5 μg/ml, or with Fc-Tag (1.9 μg/ml; adapted to the highest CCN1-Fc concentration) for control. Proliferation was quantified by CellTiter-Glo® Luminescent Cell Viability Assay and apoptosis was determined by CaspaseGlo® 3/7 Assay (both Promega) according to the manufacturer’s instructions and by measuring luminescence with an Orion II luminometer (Berthold Detection Systems, Pforzheim, Germany).

### Statistical analysis

Results were expressed as mean + SD from four independent experiments. Influence of CCN1-Fc on cell viability was calculated by nonparametric Kruskal-Wallis analysis with Dunn’s post-hoc multiple comparison test using GraphPad Prism 6.03 (GraphPad Software, CA, USA). *P* values less than 0.05 were considered to be statistically significant. Significances of qPCR analysis was calculated with the Relative Expression Software Tool (REST 2009 V2.0.13) obtained from Qiagen GmbH (Hilden, Germany) [39].

## Abbreviations

BMP: Bone morphogenetic proteins; CCN1: CCN family member 1; CCN2: CCN family member 2; CCN3: CCN family member 3; CDS: Coding sequence; CM: Conditioned media; CT: Carboxy-terminal domain; CTGF: Connective tissue growth factor; CYR61: Cysteine rich angiogenic inducer 61; DKK-1: Dickkopf-1; ECM: Extracellular matrix; EEF1A1: Eukaryotic translation elongation factor 1 alpha 1; EMT: Epithelial mesenchymal transition; ERK: Extracellular signal-regulated kinase; FCS: Fetal calf serum; IGFBP: Insulin-like growth factor binding protein; IL-6: Interleukin-6; MAPK: Mitogen-activated protein kinase; MM: Multiple myeloma; MSC: Mesenchymal stem cells; NOV: Nephroblastoma overexpressed; OPC: Osteogenic precursor cells; PBS: Phosphate buffered saline; PCR: Polymerase chain reaction; PTH1R: Parathyroid hormone 1 receptor; PVDF: Polyvinyldifluoride; RANKL: Receptor activator of NFκB ligand; RPS27A: Ribosomal protein S27a; SD: Standard deviation; SFRP2/3: Secreted frizzled related proteins 2 and 3; STAT3: Signal transducer and activator of transcription 3; SOST: Sclerostin; TBS: Tris buffered saline; TGFβ: Transforming growth factor-beta; TNFα: Tumor-necrosis factor alpha; TSR: Thrombospondin type I repeat (TSR); vWC: von Willebrand factor type C.

## Competing interest

The authors declare that they have no competing interests.

## Authors’ contributions

JD: carried out the experiments and wrote the manuscript; RE, SK and RJT: drafted the manuscript; FJ and NS: conceived the study, participated in the design of the study and drafted the manuscript. All authors read and approved the final manuscript.

## Author’s information

Senior authorship Franz Jakob and Norbert Schütze.

## Supplementary Material

Additional file 1: Figure S1 Nucleotide sequence of *CCN1* gene. *CCN1* sequence (accession number DNA sequence: NC_000001.10) including coding sequence (CDS) (underlined), parts of exon 1 and 2 (capital letters), intron 1 (gray, lower case letters) and stop codon (box). Arrows represent location of *CCN1 1*-*2* primers. Click here for file

Additional file 2: Figure S2 Splicing of *CCN1* is not induced in INA-6 cells by MSC-derived conditioned media (MSC-CM). INA-6 cells were cultured either alone (-) or with CM of three different MSC donors (+). After 24 h of incubation, INA-6 cells were harvested and splicing pattern of *CCN1* pre-mRNA was investigated by semi-quantitative PCR and intron-4 spanning primers. Both, control cells (-) and cells incubated with CM (+) expressed the intron-retaining RNA isoform. Housekeeping gene *EEF1A1* served as control. Click here for file

Additional file 3: Figure S3 Splicing of *CCN1* is a rapid, concentration-dependent event in INA-6 cells. INA-6 cells were incubated with varying concentrations of CCN1-Fc for 4 h, 8 h, or 24 h. (A) Afterwards, splicing of *CCN1* was determined by semi-quantitative PCR and by using primers for exons 4-5 (*CCN1 4*-*5*), whereas *EEF1A1* served as control. (B) Bar graphs display the relative expression of intron-free isoform, which increases with concentration but not with duration of incubation. Click here for file

Additional file 4: Figure S4 Incubation with CCN1-Fc promotes *CCN1* transcription in INA-6 cells. (A-B) INA-6 cells were incubated with Fc-Tag (control) or varying concentrations of CCN1-Fc for 24 h. After total RNA isolation, (A) semi-quantitative PCR, using primers for exons 4-5 (*CCN1 4*-*5*), and (B) qPCR analyses, using intron 4 spanning primers, were performed for *CCN1* mRNA expression. Data were normalized relative to mRNA expression levels of housekeeping gene (A) *EEF1A1* or (B) *RPS27A*. Results were normalized to Fc-Tag-incubated control cells, which were defined as 1. Results shown in (B) represent mean + SD of triplicate measurement. Click here for file
